# In Vitro Antioxidant, Antiinflammation, and Anticancer Activities and Anthraquinone Content from *Rumex crispus* Root Extract and Fractions

**DOI:** 10.3390/antiox9080726

**Published:** 2020-08-10

**Authors:** Taekil Eom, Ekyune Kim, Ju-Sung Kim

**Affiliations:** 1 Majors in Plant Resource and Environment, College of Agriculture & Life Sciences, SARI, Jeju National University, Jeju 63243, Korea; taekil7@hanmail.net; 2 College of Pharmacy, Catholic University of Daegu, Gyeongsan 38430, Korea

**Keywords:** anthraquinone, free radical scavenging, inflammatory cytokines, apoptosis, *Rumex crispus*

## Abstract

*Rumex crispus* is a perennial plant that grows in humid environments across Korea. Its roots are used in traditional Korean medicine to treat several diseases, including diseases of the spleen and skin and several inflammatory pathologies. In this study, different solvent fractions (*n*-hexane, dichloromethane, ethyl acetate, *n*-butanol, and aqueous fractions) from an ethanol extract of *R. crispus* roots were evaluated for the presence and composition of anthraquinone compounds and antioxidants by checking for such things as free radical scavenging activity, and electron and proton atom donating ability. In addition, anti-inflammatory activity was measured by NO scavenging activity and inflammatory cytokine production; furthermore, anti-cancer activity was measured by apoptosis-inducing ability. Polyphenolic and flavonoid compounds were shown to be abundant in the dichloromethane and ethyl acetate fractions, which also exhibited strong antioxidant activity, including free radical scavenging and positive results in FRAP, TEAC, and ORAC assays. HPLC analysis revealed that the dichloromethane fractions had higher anthraquinone contents than the other fractions; the major anthraquinone compounds included chrysophanol, emodin, and physcione. In addition, results of the anti-inflammatory assays showed that the ethyl acetate fraction showed appreciable reductions in the levels of nitric oxide and inflammatory cytokines (TNF-α, IL-1β, and IL-6) in Raw 264.7 cells. Furthermore, the anthraquinone-rich dichloromethane fraction displayed the highest anticancer activity when evaluated in a human hepatoma cancer cell line (HepG2), in which it induced increased apoptosis mediated by p53 and caspase activation.

## 1. Introduction

Reactive oxygen species (ROS), also known as oxygen-centered free radicals, are produced during normal metabolic processes and play an essential role in maintaining cellular homeostasis. ROS levels can increase as a result of exposure to chemical substances or other environmental stress resulting in oxidative stress [1]. When the intrinsic antioxidant system within an organism is damaged, it is not possible to remove these free radicals and the resulting oxidative stress can lead to various chronic diseases. A state of chronic oxidative stress can cause oxidative damage to various cellular components, including cell membranes, DNA, and proteins. It can also result in the activation of systemic chronic inflammatory responses via a number of different intracellular signaling pathways, ultimately exacerbating a variety of pathological conditions, including cardiovascular diseases, cancer, dementia, diabetes, autoimmune disorders, and aging [2]. One of the underlying signaling mechanisms triggered by excessive ROS generation is the activation of nuclear transcription factor κB (NF-κB), which acts as a transcriptional regulator of the innate immune system and can stimulate the release of a variety of pro-inflammatory cytokines from various tissues [3,4].

Inflammation is one of the self-defense responses used by organisms to defend against a wide range of external stimuli; however, excessive or prolonged inflammation can lead to the development of serious pathologies. The inflammatory response is characterized by the activation of macrophages and subsequent increases in the secretions of nitric oxide (NO), pro-inflammatory cytokines such as interleukin-1β (IL-1β), interleukin-6 (IL-6), and tumor necrosis factor-α (TNF-α), and cell adhesion molecules [5]. ROS-mediated chronic inflammatory responses can be inhibited by antioxidants. Various antioxidant compounds have been described in the literature with varying degrees of efficacy. The versatility of these compounds makes them ideal candidates for novel therapies. Various natural products have been shown to exhibit antioxidant properties and hence this is a growing field of interest. Plants have been identified as an especially rich source of antioxidant compounds, with most containing phenolic groups, which are known to play a crucial role in the removal of ROS. These phenolic compounds are generally secondary metabolites involved in stress responses and are known to perform various physiological functions, including antioxidant, anti-inflammatory, and anticancer functions [6,7]. The *Rumex* genus belongs to the Polygonaceae family and includes *R. crispus*, *R. acetosella, R. acetosa, R. aquatica, R. longifolius, R. gmelini, R. conglomeratus*, and *R. maritimus*. *R. crispus* is a perennial plant endemic to Korea which is found growing in humid environments. Its roots have been used as traditional medicinal materials in the treatment of several pathological conditions, including bladder infections, gallbladder disease, skin disease, and lymph node disorders. They have also been used as an adjuvant therapy in oriental medicine strategies used to treat cancer [8,9]. Several bioactive components of *R. crispus* have been identified and include saponins, tannins, flavonoids, essential oils, and anthraquinone derivatives such as chrysophanol and emodin [10,11,12]. This study was designed to evaluate the potential of new bioactive substances from *R. crispus* identified by analyzing their antioxidant, anti-inflammatory, and anticancer activities using root extracts and various extract fractions.

## 2. Materials and Methods

### 2.1. Materials

2,2-Diphenyl-1-picrylhydrazyl (DPPH), 2,2′-azobis(2-methylpropionamidine) dihydrochloride (AAPH), 2,2′-azino-bis(3-ethylbenzothiazoline-6-sulfonic acid) diammonium salt (ABTS), 2′,7′-dichlorofluorescin (DCFH), trolox, Folin-Ciocalteu reagent, 2,4,6-tris (2-pyridyl)-s-triazine (TPTZ), gallic acid, quercetin, trichloroacetic acid (TCA), aluminum chloride hexahydrate (AlCl_3_·6H_2_O), phenazine methosulphate (PMS), β-nicotinamide adenine dinucleotide reduced disodium salt (NADH), nitro blue tetrazolium tablet (NBT), thiazolyl blue tetrazolium bromide (MTT), dimethyl sulfoxide (DMSO), and lipopolysaccharide (LPS) were purchased from Sigma-Aldrich (St. Louis, MO, USA). The human hepatoma cancer cell line HepG2 and mouse macrophage cell line Raw 264.7 were obtained from the American Type Culture Collection (Manassas, VA, USA). All western blot antibodies were obtained from Santa Cruz Biotechnology (Logan, UT, USA). All other chemicals used in this study were at least 99% pure.

### 2.2. Preparation of Extracts and Fractions

Dried *R. crispus* root powder was extracted three times using ten times its weight of ethanol and subjected to reflux for 12 h. After drying by evaporation in a vacuum rotary evaporator, the extract was suspended in water and fractionated with *n*-hexane (HF), dichloromethane (DCMF), ethyl acetate (EAF), *n*-butanol, (BF), and water (AF) three times, respectively. A total of five fractions were obtained after the solvents were removed.

### 2.3. Determination of Total Phenol and Flavonoid Contents

The total phenolic content was quantified using Folin–Ciocalteu reagent and a Gallic acid standard [13]. Briefly, 100 μL of each sample solution (1 mg/mL) was mixed in a test tube containing 3.5 mL distilled water and 500 μL 50% Folin–Ciocalteu reagent. The mixture was then allowed to react for 2 h, after which 500 μL of 20% Na_2_CO_3_ was added. The mixture was then placed in a dark room for 1 h, and the absorbance at 720 nm was recorded using a SpectraMax M2^e^ microplate reader (Molecular Device, Sunnyvale, CA, USA). The total phenolic contents are expressed as gallic acid equivalents (mM GAE/g).

To analyze total flavonoid content of each sample, 500 μL of each sample (1 mg/mL) was mixed with 100 μL of 10% (*w*/*v*) aluminum chloride and 100 μL of 1.0 M potassium acetate. Then, 1.5 mL of ethanol and 2.8 mL of distilled water were added and mixed in. The mixture was then placed in a dark room for 1 h, and the absorbance at 415 nm was recorded using a SpectraMax M2^e^ microplate reader [14]. The total flavonoid content is expressed as quercetin equivalents (mM QE/g).

### 2.4. HPLC Analysis of Anthraquinone Derivative

Anthraquinone derivatives in the *R. crispus* extracts and solvent fractions were analyzed using high performance liquid chromatography coupled with a PDA detection system (Shimadzu Prominence, Japan). The analysis was performed on a Triat C-18 column (250 mm × 4.6 mm, 5 μm) from YMC Co., Ltd. The column temperature was set to 40 °C and the detection wavelength was set to 450 nm. The mobile phase consisted of water containing 0.1% TFA (Trifluoro aceticacid) and 0.1% TFA containing methanol (B) with the gradient program set as follows: isocratic 20% B at 0–5 min, linear gradient 20–80% B at 5–15 min, linear gradient 80–90% B at 15–30 min, linear gradient 90–100% B at 30–35 min, isocratic 100% B at 35–40 min with flow rate of 1.0 mL/min.

### 2.5. DPPH Radical Scavenging Activity

The DPPH radical scavenging effect was evaluated using the published method with slight modifications [15]. Briefly, 160 μL of 1.5 × 10^−4^ M DPPH solution was mixed with 40 μL of a sample solution, incubated at room temperature for 30 min, and then absorbance at 540 nm was evaluated using a SpectraMax M2^e^ microplate reader. The scavenging activity of the DPPH radicals was calculated as follows: ((Abs blank−Abs sample)/Abs blank) × 100. The radical scavenging activity was expressed as a concentration that inhibited the radicals by 50%.

### 2.6. Hydroxyl Radical Scavenging Activity

The hydroxyl radical scavenging activity was determined using the method described by Label and Bondy [16]. Briefly, the sample was mixed with 1 mM H_2_O_2_ and 0.2 mM FeSO_4_, and incubated at 37 °C for 5 min. Esterase-treated 2 μM DCHF-DA was then added and the change in fluorescence was monitored on a SpectraMax M2^e^ microplate reader, with excitation and emission wavelengths of 460 nm and 530 nm, respectively, for 30 min. The scavenging activity of the hydroxyl radicals was calculated as follows: ((FLU blank−FLU sample)/FLU blank) × 100. The radical scavenging activity was expressed as a concentration that inhibited the radicals by 50%.

### 2.7. Superoxide Radical Scavenging Activity

The superoxide radical scavenging effect was evaluated using the method reported by Liu et al. [17] with minor modifications. Briefly, the reagent mixture containing a 50 μL aliquot of a sample solution, 50 μL of 150 μM NBT, 50 μL of 468 μM NADH, and 50 μL of 60 μM phenazine methosulfate was incubated at room temperature for 5 min. The absorbance was measured at 560 nm and compared to the blank, and the superoxide anion radical scavenging activity was then calculated using the following equation: Scavenging effect, % = ((Abs sample−Abs blank)/Abs blank) × 100. The radical scavenging activity was expressed as a concentration that inhibited the radicals by 50%.

### 2.8. TEAC Assay

The TEAC method is based on the reaction of ABTS^•+^ ions and was carried out according to the method described by Zulueta et al. [18] with minor modifications. An ABTS^•+^ working solution was prepared daily by diluting the ABTS^•+^ stock solution with distilled water to get an absorbance of 0.07 ± 0.02 at 734 nm. Briefly, 50 μL aliquots of the sample solutions were each mixed with 1.0 mL ABTS^•+^ working solution. Each mixture was incubated at 25 °C in the dark for 5 min and absorbance was measured using a SpectraMax M2^e^ microplate reader at 734 nm. The sample extract activity was expressed as mM trolox/g dry sample and all determinations were carried out in triplicate.

### 2.9. ORAC Assay

ORAC measures the antioxidant inhibition of peroxyl-radical-induced oxidations and reflects radical chain-breaking antioxidant activity by H-atom transfer. This assay is based on the scavenging of peroxyl radicals generated by AAPH, which prevents the degradation of the fluorescein probe, and consequently, prevents the loss of fluorescence. For this study, we used the method described by Zulueta et al. [18]. A 75 mM phosphate buffer (pH 7.4) was used for all sample dilutions and reagent preparations. Aliquots of the sample extractions (50 μL) and the 150 μL 75 nM fluorescein solutions were placed in 96-black well microplates. The mixture was preincubated for 10 min at 37 °C. The reaction was initiated by adding 25 μL of 120 mM AAPH solution and the changes in the fluorescence were monitored using a SpectraMax M2^e^ microplate reader, with excitation and emission wavelengths of 460 nm and 530 nm, respectively, for 60 min. The sample extract activity was expressed as mM of trolox/g dry sample and all determinations were carried out in triplicate.

### 2.10. FRAP Assay

The FRAP value was determined using the method described by Benzie et al. [19] with slight modifications. Briefly, 50 μL aliquots of the sample extracts each were mixed with 1.5 mL FRAP working reagent prepared fresh daily. The FRAP working reagent consisted of 10 volumes of 300 mM acetate buffer (pH 3.6) mixed with 10 volumes of 20 mM FeCl_3_. In addition, one volume of 10 mM TPTZ in 40 mM HCl, was also added to each sample and the final mixture was incubated at 37 °C in the dark for 30 min. Absorbance was measured after 30 min at 593 nm. The activities of each extract are expressed as mM of FeSO_4_/g dry sample and all determinations were carried out in triplicate.

### 2.11. Cell Culture and Cell Viability Assays

HepG2 and Raw 264.7 cells were cultured in Dulbecco’s modified Eagle’s medium supplemented with 10% heated-inactivated fetal bovine serum, penicillin (100 U/mL), and streptomycin (100 μg/mL) at 37 °C in a humidified atmosphere of 95% air and 5% CO_2_. The medium was changed every other day. Cell viability was measured using the MTT assay, which is based on the conversion of MTT to formazan crystals by mitochondrial dehydrogenases. Cells were cultured in 96-well plates (2.0 × 10^4^ cells/well) with serum free media and treated with different concentrations of sample for 24 h. The *R. crispus* extracts and its solvent fractions were dissolved in 10% DMSO. The final concentration of DMSO in the culture medium never exceeded 0.1%. For the assay, 100 μL of MTT solution was added to each well and incubated for 4 h. Finally, 200 μL of DMSO was added to dissolve the formazan crystals and the absorbance was measured using a SpectraMax M2^e^ microplate reader at 540 nm.

### 2.12. NO Production

Raw 264.7 cells were cultured in 96-well plates using media without phenol red and pre-treated for 1 h with each of the test substrates. Cellular NO production was induced by adding 1 μg/mL LPS and incubating the mixture for 24 h. After incubation, 50 μL of conditioned media containing nitrite (primary stable oxidation product of NO) was mixed with the same volume of Griess reagent and incubated for 15 min. Absorbance of the mixture was measured using a SpectraMax M2^e^ microplate reader at 550 nm.

### 2.13. Cytokine Analysis

Production of IL-1β, IL-6, and TNF-α in Raw 264.7 cells was evaluated using Quantikine ELISA kits (R&D Systems, Minneapolis, MN, USA) as per the manufacturer’s instructions. Cells were treated with different concentrations of test materials for 1 h and production of IL-1β, IL-6, and TNF-α was stimulated by adding 1 μg/mL LPS and incubating for a further 24 h. The supernatant was collected and the concentrations of IL-1β, IL-6, and TNF-α were quantified using the relevant kit protocol.

### 2.14. Annexin V-FITC/PI Analysis

To determine the magnitude of the apoptosis induced by DCMF, an Annexin V-fluorescein isothiocyanate (FITC) apoptosis detection kit (BD Pharmingen, San Diego, CA, USA) was used. Briefly, the cells were harvested, washed with PBS and binding buffer, and then stained with FITC-conjugated Annexin V and propidium iodide (PI) for 30 min in the dark. The mixture was then analyzed using an LSR Fortessa flow cytometer (Becton Dickinson, San Jose, CA, USA) according to the manufacturer’s protocol.

### 2.15. Western Blot

HepG2 cells were cultured in DMEM at a density of 1 × 10^4^ cells in 10 cm^2^ cell culture dishes and incubated for 24 h. The cells were treated with different concentrations of DCMF for 24 h. The cells were lysed using RIPA buffer (Sigma-Aldrich, St. Louis, MO, USA) and supernatants were treated with a protease inhibitor cocktail and centrifuged at 2300× *g* for 10 min to remove the insoluble fraction. The protein concentrations of the supernatants were determined using a BCA protein assay kit (Thermo Science, Rockford, IL, USA).

The same amounts of cell lysates were analyzed on 10% SDS-PAGE and the proteins were blotted onto immuno-blot nitro-cellulose membranes and blocked with 5% BSA in TBS containing 0.1% Tween 20 (TBS-T) for 1 h. Then the primary monoclonal antibodies were added to the TBS-T (1:1000 dilutions) and incubated overnight. Antibody binding was detected using a horseradish peroxidase secondary antibody and enhanced using a chemi-luminescence ECL assay kit (Bio-Rad, Hercules, CA, USA) according to the manufacturer’s instructions and imaged on a FUJIFILM LAS-4000 mini system (Tokyo, Japan). The basal levels of the proteins were normalized against β-actin or β-tubulin.

### 2.16. Statistical Analysis

Each experiment was performed at least three times and results are presented as means ± SDs (standard deviations). Statistical comparisons of the mean values were performed using one-way ANOVA followed by Duncan’s multiple range test using Minitab 17 software (Minitab Inc., IL, USA, State College, PA, USA). Differences were considered significant at *p* < 0.05.

## 3. Results and Discussion

### 3.1. Analysis of Polyphenol, Flavonoid, and Anthraquinone Contents

Polyphenols are aromatic compounds containing more than two phenolic hydroxyl groups. They are classified into phenolic acids (e.g., caffeic acid and chlorogenic acid) and flavonoids (e.g., kaempferol and catechin) [20]. The total polyphenol and flavonoid contents for each of the extracts are described in Table 1. Analysis of the total polyphenol and flavonoid content in the *R. crispus* extracts and solvent fractions revealed that polyphenol content was highest in the ethyl acetate fraction (EAF), followed by the dichloromethane fraction (DCMF), ethanol extract (EE), aqueous fraction (AF), butanol fraction (BF), and finally, the hexane fraction (HF). The highest flavonoid content was detected in the DCMF, followed by HF, EAF, EE, AF, and BF. The antioxidant activity of polyphenolic compounds is attributed to their activities as electron donors and free radical scavengers. Therefore, the antioxidant effects of various plant extracts have been shown to be strongly linked with the relative phenolic content [21,22].

An HPLC-DAD method was applied to identify the five anthraquinones, including aloeemodin, chrysophanol, emodin, physcion, and rhein, in *R. crispus* extracts and solvent fractions. Figure S1 shows the typical chromatograms of the standard solution containing the five anthraquinones and the *R. crispus* extracts and solvent fractions. The retention times of the aloeemodin, rhein, emodin, chrysophanol, and physcion were 22.2, 23.7, 27.3, 29.9, and 33.2 min, respectively (Figure S1). The concentrations of the major anthraquinones from *R. crispus* extracts and solvent fraction are summarized in Table 2. The major anthraquinones found in the samples analyzed in this study were chrysophanol, emodin, and physcion. The anthraquinone content was highest in the DCMF, followed by HF, EE, EAF, and BF. One gram of DCMF contained 66.96 mg chrysophanol, 160.43 mg emodin, and 34.90 mg physcion. One gram of HF contained 48.64 mg chrysophanol, 14.64 mg emodin, and 15.43 mg physcion. However, none of the anthraquinone compounds were detected in the AF. Anthraquinone derivatives are naturally occurring quinone compounds including naphthoquinones and benzoquinones, and are present in large quantities in plants such as Polygonaceae (*Rheum*, *Rumex*), Fabaceae (*Cassia*), Liliaceae (*Aloe*), Rhamnaceae (*Rhamnus*), and Rubiaceae (*Asperula*, *Coelospermum*, *Coprosma*, *Galium*, *Morinda*, and *Rubia*) [23]. Lim et al. [24] analyzed the anthraquinone contents of various *Rumex* species and found that emodin was highest in *R. crispus*. In addition, Smolarz et al. [25] investigated the anthraquinone contents of various *Rumex* species and found that the highest anthraquinone concentrations were found in the root extracts, with these extracts having substantially higher concentrations than those of the fruit extracts (70-fold) and the leaf extracts (10-fold). Most of these compounds are nonpolar with a 9,10-anthracenedione basic structure—a tricyclic aromatic organic compound with a formula of C_14_H_8_O_2_, which is extracted by polar solvents like ethanol/water mixtures, ethanol, methanol, and acetone [26]. It has also been reported that these compounds are well dispersed in nonpolar solvents, such as hexane and dichloromethane [27].

### 3.2. Radical Scavenging Activities of R. crispus Extracts and Fractions

The DPPH radical scavenging assay is used to assess the electron-donating ability of antioxidants to quench free radicals. In *R. crispus* root extracts and fractions, we observed that DPPH radical scavenging was the highest in the EAF, followed by the EE, BF, DCMF, and HF (Table 3). While the antioxidant activities of *R. crispus* leaf and fruit extracts have been extensively studied, there is limited information on these activities in its root extracts. Yildirim et al. [28] have reported that DPPH radical scavenging activity is higher in *R. crispus* fruit extracts with high polyphenol content than in leaf extracts. Consistent with the findings of this study, the radical scavenging ability of *R. japonica* extracts and fractions has also been found to be highest in extracts with high polyphenol content and low in extracts with low polyphenol content [29].

We measured the scavenging capacities of these extracts for hydroxyl radicals using the Fenton reaction assay, which is based on fluorescence emission after hydroxyl radicals generated by H_2_O_2_ and Fe^2+^ via the Fenton reaction with DCFH. The scavenging ability increased in all the extracts and fractions in a concentration-dependent manner. This is similar to the results for Trolox, a well-known antioxidant. Anusuya et al. [30] have reported that the hydroxyl radical scavenging abilities of *Rubus nepalensis* extracts are closely related to their polyphenol contents. Moreover, phenolic hydroxyl groups are known to rapidly quench hydroxyl radicals by donating hydrogen atoms or electrons, as evidenced by measuring the hydroxyl radical scavenging abilities of various phenolic acids [31]. This study also demonstrated that the EAF and DCMF which both had high polyphenol and flavonoid contents also had the highest hydroxyl scavenging abilities (Table 3).

Superoxide radicals have very low reactivity; however, within the body, they are rapidly transformed into H_2_O_2_, and then via the Fenton reaction, to highly reactive hydroxyl radicals, which interact with biomolecules, causing tissue damage. As with hydroxyl radical scavenging, the superoxide radical scavenging ability of *R. crispus* root extracts was strongest in the EAF and DCMF, followed by EE, BF, AF, and HF (Table 3). In a study of the antioxidant properties of *Rumex hastatus* extracts and fractions, superoxide radical scavenging ability has been linked to flavonoid content rather than phenolic content [32]. The present study showed that the EAF and DCMF fractions, with high flavonoid content, exhibited the highest superoxide radical scavenging ability.

### 3.3. Antioxidant Capacities of R. crispus Extracts and Fractions

The FRAP assay determines antioxidant activity by measuring electro transport. In this assay, the antioxidant activity was assessed by the reduction of Fe^3+^-TPTZ to Fe^2+^-TPTZ. The FRAP value of the extracts and fractions was the highest in the EAF, followed by the EE, AF, DCMF, BF, and HF (Table 4). The TEAC assay measures scavenging of ABTS^+^ radicals by sulfur oxides through donation of hydrogen atoms or electrons, which are expressed as trolox equivalents. The highest electron-donating capacity was recorded for the EAF (5.65 mM TE/g), followed by the DCMF, EE, AF, BF, and HF (Table 4). The ORAC assay is an experimental method recommended by the United States Department of Agriculture for the quantification of food antioxidant content based on electron transport capacity. Decomposition of AAPH produces peroxyl radicals, which react with fluorescein, leading to decreased fluorescence. Peroxyl radical scavenging measured as fluorescence reduction is then converted to trolox equivalents (TE). The ORAC value was highest in the EAF (4817 mM TE/g) and decreased in order from DCMF, to EE, then HF, then AF, and finally BF (Table 4).

The antioxidant capacities of *R. crispus* were highest in the EAF and DCMF, which is consistent with their total phenol and flavonoid contents. Similarly, the antioxidant activity of *R. hastatus* extracts has been reported to be highest in the EAF which has high total phenol and flavonoid concentrations [32]. Sahidi and Ambigaipalan [33] have reported that food antioxidant capacity is closely related to the total phenolic and flavonoid contents and have attributed it to the electron or H-atom-donating ability of phenolic hydroxyl groups. In this study, the antioxidant capacities were highest in the EAF and DCMF, which is consistent with their higher concentrations of phenolic and flavonoid compounds.

### 3.4. Anti-Inflammation Activities of R. crispus Extracts and Fractions

**Figure 1:**
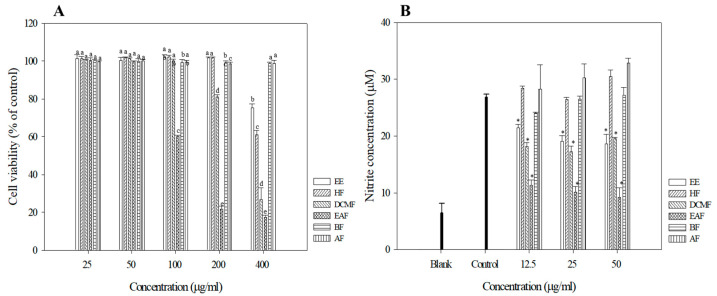
Cell viabilities of *R crispus* extracts and solvent fractions. (**A**) NO production (**B**) in Raw 264.7 cells. RAW 264.7 cells were treated with various concentrations (25, 50, 100, 200, 400 μg/mL) of *R. crispus* extracts and fractions for 24 h. Cell viability was measured by MTT assay. RAW 264.7 cells were pre-incubated with 12.5, 25, and 50 μg/mL of extracts and fractions for 1 h and then treated with 1 μg/mL of LPS for 24 h. The NO production was measured by the Griess reagent system. Data are represented as means ± SEMs. The different superscripts are significantly different at *p* < 0.05. * Statistical significance of the difference between LPS and LPS + sample treatment groups: * *p* < 0.05.

To assess the anti-inflammatory activities of *R. crispus* extracts and fractions, we measured their cytotoxicities in mouse leukemic monocyte macrophage cells (Raw 264.7). Raw 264.7 cells were treated with 25–400 μg/mL of *R. crispus* extracts and fractions, and cell survival was measured after 24 h using the WST-1 assay. Cell viabilities of *R. crispus* extracts and fractions are summarized in Figure 1A. The EE, BF, and AF did not show cytotoxicity within the indicated concentration range. The EE and HF were not cytotoxic at a concentration of 25–200 μg/mL, but at 400 µg/mL cell survival decreased to 75.24% and 60.94%, respectively. The EAF showed the highest cytotoxicity, decreasing cell survival to 60.20%, 2.62%, and 17.33% at 100, 200, and 400 μg/mL, respectively (Figure 1A).

Excessive NO production from L-arginine due to an overexpression of inducible NO synthase (iNOS) during acute or chronic inflammation is known to accelerate inflammatory responses [34]. We assessed the inhibition of NO production induced by LPS at non-cytotoxic concentrations (25 and 50 µg/mL) for each of the extract fractions. Consistent with the results of the antioxidant assays the EAF and DCMF showed the highest inhibitions of NO production, while other fractions did not exhibit any inhibitory effects (Figure 1B). Previous studies on the anti-inflammatory activities of *R. crispus* leaf extracts and fractions have also reported high levels of inflammatory activity in the DCMF and EAF, which agrees with the findings of this study [35]. Based on the results of the NO inhibition assay, we used the EAF for further cell-based experiments.

**Figure 2:**
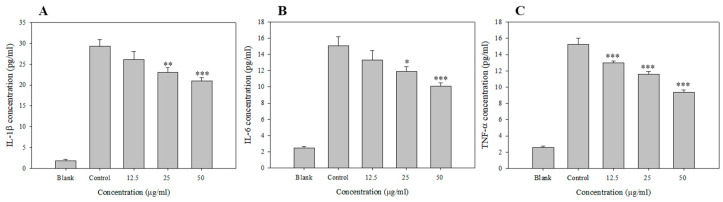
Inhibition of LPS induced IL-1b, IL-6 and TNF-a in the EAF. RAW 264.7 cells were preincubated with 12.5 or 25 μg/mL of EAF for 1 h and then treated with 1 μg/mL of LPS for 24 h. The IL-1β, IL-6, and TNF-α production was measured by ELISA, as described in Materials and Methods. Data are represented as means ± SEMs. * Statistical significance of the difference between LPS and LPS + sample treatment groups: * *p* < 0.05, ** *p* < 0.01, *** *p* < 0.001.

Cytokines play a pivotal role in inflammatory responses by directly affecting the proliferation and activity of immune cells [36]. Among the pro-inflammatory cytokines, TNF-α is an essential mediator in the development of systemic inflammatory responses, and its synthesis is increased by NO generated by LPS-stimulated macrophages. TNF-α increases the expressions of chemokines and cell adhesion molecules, thereby accelerating pro-inflammatory responses [37]. IL-1β and IL-6 are multifunctional cytokines secreted by macrophages activated by various pro-inflammatory stimuli; these cytokines are also implicated in the induction of autoimmune diseases, and they act by accelerating inflammatory responses through autocrine signaling [38]. Figure 2 shows the inhibition of IL-1β, IL-6, and TNF-α secretion following EAF treatment using the enzyme-linked immunosorbent assay (ELISA). The secretion of pro-inflammatory cytokines by Raw 264.7 cells was sharply increased following LPS stimulation and decreased after treatment with EAF in a concentration-dependent manner. IL-1β, IL-6, and TNF-α were suppressed by 28%, 65%, and 68%, respectively, when treated with 50 μg/mL EAF.

In a study of the anti-inflammatory activity in *R. crispus* leaf extracts and fractions, Im et al. [35] found that the EAF displayed higher concentration-dependent activity than the extracts in inhibiting the expression of COX-2 and iNOS involved in the production of PGE_2_ and NO. In this study, the anti-inflammatory effects were verified by the reduced expression of all the pro-inflammatory cytokines apart from TNF-α [39].

### 3.5. Anticancer Activities of R. crispus Extracts and Fractions

**Figure 3:**
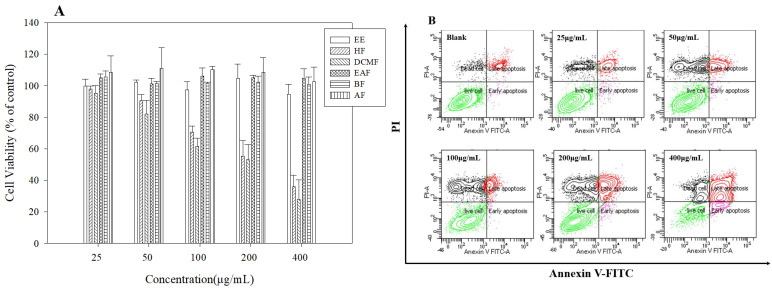
Cell viability of *R crispus* extracts and solvent fractions. (**A**) apoptosis induced (**B**) in HepG2 cells. HepG2 cells were treated with various concentrations (25, 50, 100, 200, 400 μg/mL) of *R. crispus* extracts and fractions for 24 h. Cell viability was measured by MTT assay. Flow cytometry analysis of apoptosis after exposure to various concentrations (25, 50, 100, 200, 400 μg/mL) of DCMF for 24 h, using annexin V-FITC/PI. The lower right indicates the percentage of early apoptotic cells; the upper right indicates the percentage of necrotic and late apoptotic cells.

We measured the cytotoxic activities of the fractions against the HepG2 human hepatoma cancer cell line. Among the extracts and fractions, anthraquinone-rich fractions HF and DCMF appeared to be the most potent inhibitors of HepG2 cell proliferation, but the other fractions showed no cytotoxicity. The DCMF inhibited cell growth in a dose-dependent manner. Treatment with HF for 24 h inhibited cell viability with rates of approximately 97%, 90%, 70%, 55%, and 35% at concentrations of 25, 50, 100, 200, and 400 μg/mL, respectively. Treatment with DCMF for 24 h resulted in inhibition of cell viability with rates of approximately 95%, 82%, 61%, 53%, and 27% at concentrations of 25, 50, 100, 200, and 400 μg/mL, respectively (Figure 3A).

A previous study has shown that anticancer activities of *Rumex* species are closely related to their anthraquinone contents [8,40]. In this study, anticancer activity was highest in the HF and DCMF, which is consistent with their anthraquinone content. Based on these results we used the DCMF for further cell-based experiments. As a decrease in cell proliferation may result from the induction of apoptosis, we investigated whether treatment with the DCMF induced apoptosis in HepG2 cells. HepG2 cells were treated with DCMF at various concentrations; then Annexin V-FITC and PI fluorescence was determined by flow cytometry (Figure 3B). After treatment with 50, 100, 150, and 200 μg/mL DCMF for 48 h, the percentages of apoptotic cells were 13.3%, 15.9%, 25.6%, and 71.3%, respectively. These results suggest that the DCMF inhibited the proliferation of HepG2 cells by inducing apoptosis in a concentration-dependent manner.

### 3.6. Modulation of Apoptotic Regulation

**Figure 4:**
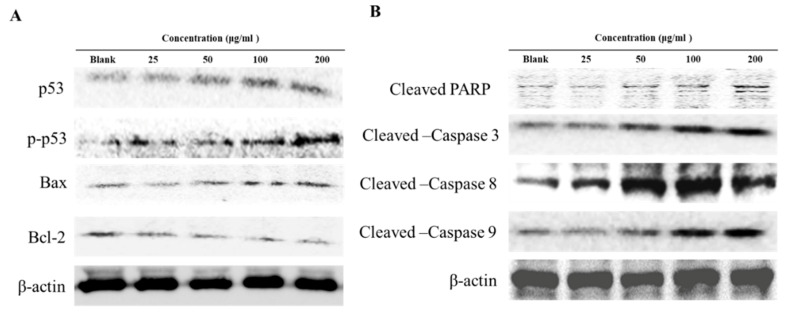
Effects of DCMF on the Bcl-2 family and p53 (**A**) and caspase family (**B**) protein expression in HepG2 cells. HepG2 were treated with the indicated concentrations of DCMF for 24 h. The equal amounts of cellular proteins were probed with the indicated antibodies, and the proteins were visualized using an ECL detection system. Actin was used as an internal control.

Bcl-2 proteins play a complex regulatory role in apoptosis [41]. Treatment with DCMF resulted in decreased Bcl-2 expression, while Bax protein expression was increased in a dose dependent manner. DCMF showed increased p53 tumor suppressor protein expression in HepG2 cells (Figure 4A). These results indicate that part of the DCMF-mediated inhibition of HepG2 cells is related to apoptosis through its effects of p53 and Bcl-2 protein expression. In order to determine whether this inhibition is related to the induction of apoptosis, HepG2 cells were exposed to DCMF and their caspase activity was evaluated. DCMF treatment resulted in increased levels of cleaved caspase-3, -8, and -9. This result was confirmed by the progressive proteolytic cleavage of the poly (ADP-ribose) polymerase (PARP), a downstream target of activated caspase, in HepG2 cells treated with DCMF (Figure 4B). These data agree with the other experiments that suggest that DCMF treatment induces apoptosis in HepG2 cells.

DCMF-induced apoptosis in HepG2 cell was confirmed by the characteristic pattern of Annexin V/PI staining, activation of caspases (-3, -8, and -9), and cleavage of PARP. Activated caspases regulate the execution-phases of cell apoptosis by degrading specific structural, regulatory, and DNA repair proteins within the cell [42]. Activated caspase-9 then initiates the proteolytic activity of other downstream caspases, such as caspase-3. The activation of caspase-3 results in the cleavage of key cellular proteins, such as PARP [43]. Tumor suppressor protein p53 also plays key roles in cell fate, cell growth, and death, via the regulation of the cell cycle proteins [44] and apoptosis induced proteins (Apaf1, Bad, Bax, and Fas) [45]. Treatment with DCMF decreased anti-apoptotic Bcl-2 protein and increased pro-apoptotic Bax protein expression. In addition, DCMF treatment increased p53 tumor suppressor protein levels. These results demonstrate that DCMF-induced apoptosis in HepG2 human hepatoma cancer cells is affected via the induction of p53, activation of Bax, inhibition of Bcl-2, processing of caspases, and cleavage of PARP.

## 4. Conclusions

Several studies have demonstrated that natural polyphenol-containing products reduce ROS which are risk factors for age-related diseases. This study examined whether *R. crispus* extracts and fractions could exert any inhibitory effects on oxidative stress-related reactions and inflammation in vitro. The ethanol extracts of *R. crispus* were separated into hexane, dichloromethane, ethyl acetate, butanol, and aqueous fractions based on polarity. Antioxidant activity was evaluated using various assays and was shown to be highest in the DCMF and EAF, corresponding to their high polyphenol and flavonoid contents. In addition, the anti-inflammatory tests revealed that high antioxidant activity correlated with inhibitory effects on NO production, and that the EAF also reduced the secretion of pro-inflammatory cytokines in a concentration-dependent manner. In addition, DCMF was shown to inhibit HepG2 human hepatoma cancer cell growth and induce cellular apoptosis. DCMF-induced apoptosis is facilitated by p53 tumor suppressor protein-mediated Bcl-2 family protein regulation and caspase family protein activation. The results of this study suggest that *R. crispus* could be used as a natural alternative to synthetic antioxidants and anti-inflammatory agents.

## Figures and Tables

**Table 1:** Total phenolic and flavonoid contents of *Rumex crispus* L. root extracts and fractions ^1^.

Samples ^2^	Total Phenols (mg GAE/g)	Total Flavonoids (mg QE/g)
EE	21.84 ± 1.15 ^c^	14.58 ± 0.61 ^d^
HF	10.68 ± 0.06 ^e^	24.15 ± 0.47 ^b^
DCMF	28.16 ± 1.42 ^b^	30.67 ± 0.97 ^a^
EAF	83.26 ± 2.49 ^a^	21.31 ± 0.33 ^c^
BF	19.03 ± 1.04 ^c,d^	11.28 ± 0.40 ^e^
AF	19.79 ± 0.32 ^c,d^	11.52 ± 0.70 ^e^

^1^ Values are each expressed as a mean ± SD (*n* = 3). ^2^ EE: ethanol extracts. HF: *n*-hexane fractions. DCMF: dichloromethane fraction. EAF: ethyl acetate fraction. BF: *n*-buthanol fractions. AF: aqueous fraction. ^a–e^ Means with different superscripts in the same column are significantly different at *p* < 0.05.

**Table 2:** Anthraquinone derivative contents of *Rumex crispus* L. root extracts and fractions ^1^.

Samples ^2^	Concentration (mg/g)					
	Aloeemodin	Chrysophanol	Emodin	Physcion	Rhein	Total
EE	0.141 ± 0.002 ^c^	9.714 ± 0.02 ^c^	8.779 ± 0.011 ^d^	4.282 ± 0.006 ^c^	0.057 ± 0.002 ^d^	22.97 ± 0.026 ^c^
HF	0.048 ± 0.006 ^e^	48.644 ±0.171 ^b^	14.64 ± 0.037 ^b^	15.433 ± 0.058 ^b^	0.106 ± 0.001 ^c^	79.095 ± 0.259 ^b^
DCMF	0.595 ± 0.003 ^a^	66.964 ± 0.244 ^a^	160.434 ± 0.651 ^a^	34.896 ± 0.109 ^a^	0.466 ± 0.002 ^a^	263.356 ± 0.666 ^a^
EAF	0.218 ± 0.001 ^b^	3.154 ± 0.009 ^d^	13.627 ± 0.053 ^c^	1.722 ± 0.006 ^d^	0.151 ± 0.002 ^b^	18.923 ± 0.062 ^d^
BF	0.181 ± 0.007 ^d^	0.083 ± 0.002 ^e^	0.639 ± 0.004 ^e^	0.054 ± 0.002 ^e^	0.001>	0.856 ± 0.006 ^e^
AF	-	-	-	-	-	-

^1^ Values are each expressed as a mean ± SD (*n* = 3). ^2^ EE: ethanol extracts. HF: *n*-hexane fractions. DCMF: dichloromethane fraction. EAF: ethyl acetate fraction. BF: *n*-buthanol fractions. AF: aqueous fraction. ^a–e^ Means with different superscripts in the same column are significantly different at *p* < 0.05.

**Table 3:** Free radical scavenging activity of *Rumex crispus* L. root extracts and fractions ^1^.

Samples ^2^	EC_50_^3^		
	DPPH Radical	Hydroxyl Radical	Superoxide Radical
EE	46.5 ± 2.6 ^b^	19.65 ± 0.64 ^c^	51.72 ± 2.00 ^c^
HF	126.2 ± 1.3 ^e^	62.47 ± 2.44 ^f^	>200 ^f^
DCMF	65.6 ± 1.2 ^d^	0.54 ± 0.13 ^a^	45.83 ± 2.00 ^b^
EAF	11.9 ± 2.5 ^a^	0.65 ± 0.06 ^a^	4.45 ± 0.42 ^a^
BF	55.1 ± 1.5 ^c^	3.84 ± 0.35 ^d^	61.00 ± 2.81 ^d^
AF	44.2 ± 3.4 ^b^	43.12 ± 0.00 ^e^	71.29 ± 2.39 ^e^

^1^ Values are expressed as a mean ± SD (*n* = 3). ^2^ EE: ethanol extracts. HF: *n*-hexane fractions. DCMF: dichloromethane fraction. EAF: ethyl acetate fraction. BF: *n*-buthanol fractions. AF: aqueous fraction. ^3^ Effective concentration of substance that causes 50% inhibition. ^a–e^ Means with different superscripts in the same column are significantly different at *p* < 0.05.

**Table 4:** FRAP, TEAC, and ORAC values of *Rumex crispus* L. root extracts and fractions ^1^.

Samples ^2^	FRAP (mM FeSO_4_/g)	TEAC (mM TE/g)	ORAC (mM TE/g)
EE	48.14 ± 0.47 ^b^	2.46 ± 0.11 ^c^	1396 ± 204 ^b,c^
HF	13.66 ± 0.15 ^e^	0.43 ± 0.06 ^f^	983 ± 88 ^c,d^
DCMF	37.74 ± 0.77 ^c,d^	3.56 ± 0.11 ^b^	1790 ± 246 ^b^
EAF	135.58 ± 3.62 ^a^	5.65 ± 0.00 ^a^	4817 ± 331 ^a^
BF	33.06 ± 0.80 ^d^	1.97 ± 0.13 ^d^	909 ± 121 ^d^
AF	40.83 ± 0.16 ^c^	2.30 ± 0.13 ^c^	945 ± 87 ^d^

^1^ Values are expressed as a mean ± SD (*n* = 3). ^2^ EE: ethanol extracts. HF: *n*-hexane fractions. DCMF: dichloromethane fraction. EAF: ethyl acetate fraction. BF: *n*-buthanol fractions. AF: aqueous fraction. ^a–e^ Means with different superscripts in the same column are significantly different at *p* < 0.05.

## Supplementary Materials

The following are available online at https://www.mdpi.com/2076-3921/9/8/726/s1, Figure S1: Chromatogram of *Rumex crispus* root extract and fractions at 420 nm. a: Aloe-emodin, b: Rhein, c: Emodin, d: Chrysophanol. e: Physcion (EE).
