# Supplementary material for: In Vitro Antioxidant, Antiinflammation, and Anticancer Activities and Anthraquinone Content from Rumex crispus Root Extract and Fractions
Source: Antioxidants (Basel). 2020 Aug 10;9(8):726. doi: 10.3390/antiox9080726 (PMC7464605; doi:10.3390/antiox9080726)
Supplement: Supplementary file 1 [file antioxidants-09-00726-s001.pdf]

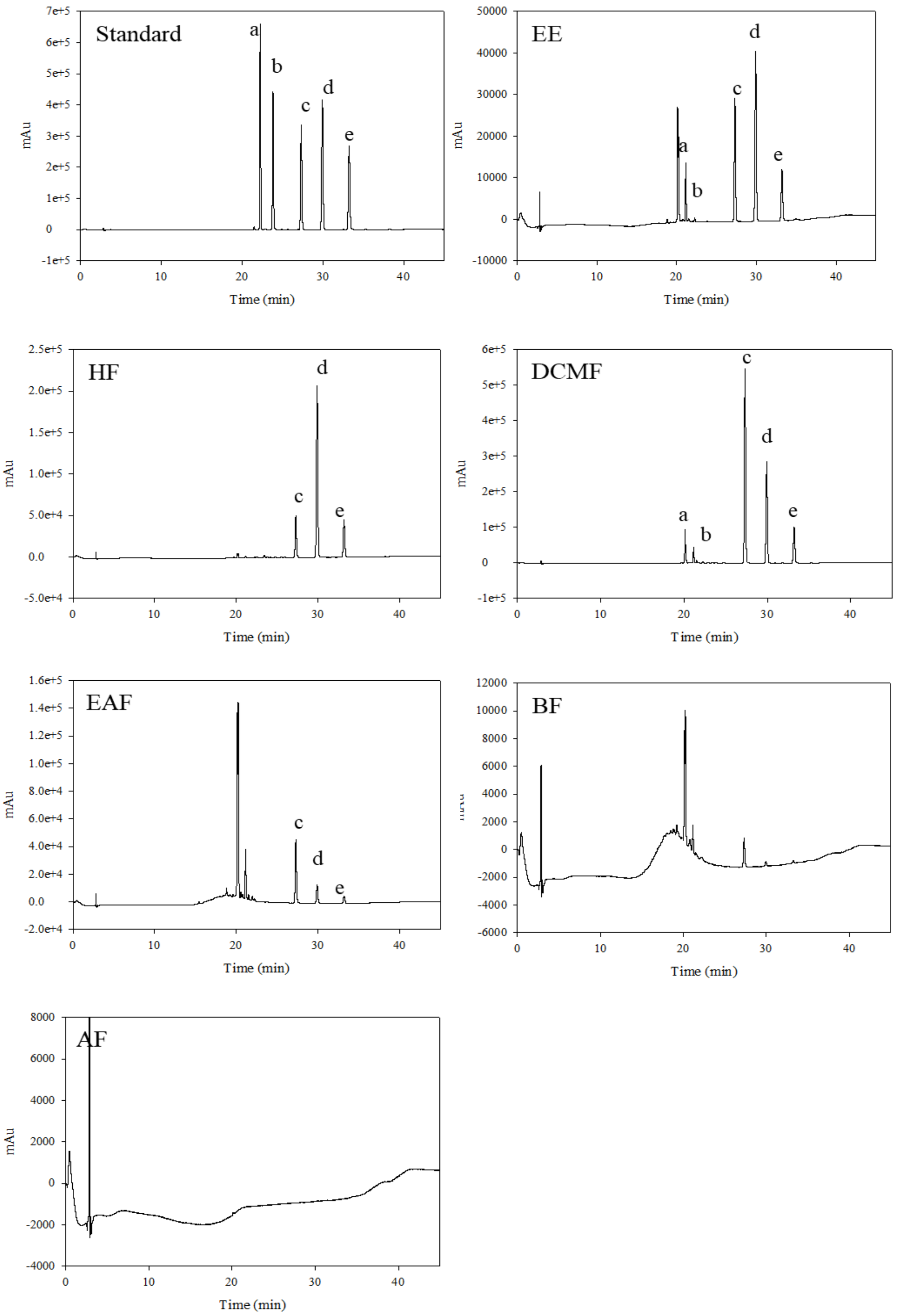

Figure S1. Chromatogram of *Rumex crispus* root extract and fractions at 420 nm. a: Aloe-emodin, b: Rhein, c: Emodin, d: Chrysophanol. e: Physcion (EE).
